# Late Growth and Changes in Body Composition Influence Odds of Developing Retinopathy of Prematurity among Preterm Infants

**DOI:** 10.3390/nu12010078

**Published:** 2019-12-27

**Authors:** Ellen C. Ingolfsland, Jacob L. Haapala, Lauren A. Buckley, Ellen W. Demarath, Sixto F. Guiang, Sara E. Ramel

**Affiliations:** 1 Department of Pediatrics, University of Minnesota, Minneapolis, East Building 6th floor, MB630, 2450 Riverside Ave, Minneapolis, MN 55454, USA; 2 Division of Epidemiology and Community Health, University of Minnesota, 300 West Bank Office Building, 1300 S 2nd St, Minneapolis, MN 55454, USA

**Keywords:** prematurity, body composition, adiposity, postnatal growth, retinopathy of prematurity

## Abstract

Background: While postnatal growth in the first month of life is known to impact retinopathy of prematurity (ROP) risk, the impact of growth later in hospitalization, during critical times of retinal vascularization, remains unknown. The purpose of this study was to assess if postnatal growth and body composition during the second half of neonatal intensive care unit hospitalization were associated with severity of retinopathy of prematurity in very low birth weight preterm infants. Methods: Prospective observational pilot study of 83 infants born <32 weeks gestation and <1500 g, conducted at a Level IV neonatal intensive care unit. Body composition was measured during the second half of hospitalization. Infants were evaluated for retinopathy of prematurity. Logistic regression was performed. Results: Greater gains in fat mass, fat-free mass, and percent body fat from 32 to 37 weeks postmenstrual age and higher % body fat at term postmenstrual age were associated with decreased odds of ≥stage 2 retinopathy of prematurity (*p* < 0.05). Conclusions: Improved growth later in neonatal intensive care unit hospitalization and increased adiposity at term may reduce odds of severe retinopathy of prematurity.

## 1. Introduction

Retinopathy of prematurity remains a significant cause of childhood blindness worldwide, and with the improving survival of preterm infants, the prevalence is likely to increase [1]. This signifies the need for a better understanding of retinopathy of prematurity pathogenesis. The impact of poor growth on developing retinal vessels is emerging with the discovery that poor postnatal growth increases risk for severe disease [2]. WINROP (weight, insulin-like growth factor 1 (IGF-1), neonatal retinopathy of prematurity risk algorithm) established that early weight gain and IGF-1 levels, primarily in the first 4 weeks of life, predict which infants will develop vision-threatening retinopathy of prematurity [2,3]. Similarly, greater early macronutrient intake and growth velocity with higher insulin-like growth factor 1 (IGF-1) and insulin-like growth factor binding protein 3 (IGFBP3) levels have been associated with decreased retinopathy of prematurity severity [4,5,6,7].

While early growth has been shown to reduce risk, the effect of later growth, nutrition, and associated growth factors on retinopathy of prematurity severity has not been determined. Moreover, the impact of later changes in body composition (fat-free mass, fat mass, and % body fat) on retinopathy of prematurity severity has also never been studied. As retinopathy of prematurity is a biphasic disease, with early retinal vasoattenuation followed by later neovascularization, we suspect that later changes in body composition may impact the neovascular phase. Adiponectin, an adipocytokine positively associated with growth in preterm infants, and IGF-1 have differential effects on the developing retina during the early vasoattenuative phase compared to the later neovascular phase of retinopathy of prematurity development [6,8,9,10,11]. Fat mass, fat-free mass, and percent body fat are differentially associated with adiponectin, IGF-1, and IGFBP3, so changes in these variables during the neovascular phase may alter retinopathy of prematurity risk [12,13,14].

The aim of this study was to test the hypothesis that faster gains in fat mass, fat-free mass, and % body fat after 29 weeks postmenstrual age and higher serum IGF-1, IGFBP3, and adiponectin levels at 7 days of life and 35 weeks postmenstrual age would decrease odds of ≥stage 2 retinopathy of prematurity among very low birth weight preterm infants.

## 2. Materials and Methods

### 2.1. Research Subjects

We conducted an observational prospective cohort study, which collected data on 103 infants at the University of Minnesota Masonic Children’s Hospital neonatal intensive care unit (NICU) from April 2011 to June 2016. The goal of this pilot study was to investigate the relationship between nutrition and longitudinal body composition changes of very low birth weight preterm infants and both short- and long-term neurodevelopmental outcomes. Inclusion criteria included gestational age at birth <32 weeks, birthweight <1500 g, and size appropriate for gestational age (between the 10th and 90th percentile on the Fenton growth chart). Small for gestational age infants were excluded due to small sample size in this pilot study to allow for analysis of similar infants without influence of growth restriction. Infants were also excluded if born with a congenital anomaly that would affect growth, enrolled in an interventional research study, or the consenting parent was a non-English speaker (interpreters were not available at follow-up study visits). Written informed consent from a parent was obtained in the first 5 days of life. Approval was granted by the University of Minnesota Institutional Review Board. Collection and maintenance of patient data was in compliance with HIPAA regulations.

### 2.2. Measurements

Body composition (fat-free mass, fat mass, and % body fat) was serially measured with air displacement plethysmography after 29 weeks postmenstrual age and at term postmenstrual age. Infants were measured when off positive pressure respiratory support and total parenteral nutrition. All infants were evaluated for retinopathy of prematurity per unit standard by a pediatric ophthalmologist starting at 31 weeks postmenstrual age. Severity of ROP was classified according to the revised International Classification of Retinopathy of Prematurity and the most severe stage during hospitalization recorded [15]. For the purpose of this analysis, retinopathy of prematurity was categorized by stage as either ≥stage 2 or <stage 2 due to small sample size and thus the small number of infants with Type 1 retinopathy of prematurity. Infants with no retinopathy of prematurity were categorized as <stage 2. As first week nutrition is critical for long term growth and neurodevelopment as well as ROP outcomes, we also measured total kcal/kg and total protein (g)/kg for each patient from day of life 2–8 [7,16,17]. In a subset of 39 patients, serum IGF-1, IGFBP3, and total adiponectin levels were obtained at 7 days and 35 weeks postmenstrual age and measured by ELISA (R&D Systems, Minneapolis, MN, USA) in the University of Minnesota Cytokine Lab.

### 2.3. Statistical Analysis

Descriptive statistics (means, medians, and frequencies) for patient characteristics were calculated. Outliers were identified in IGF-1 measurements, so to limit their influence, the top and bottom values were winsorized. Multivariate logistic regression was performed to test for associations between body composition predictors (fat mass, fat-free mass, and % body fat) at term, body composition rate of change predictors, growth factors predictors (IGF-1, IGFBP3, and adiponectin at 7 days and 35 weeks postmenstrual age), and the binary outcome (retinopathy of prematurity ≥stage 2/retinopathy of prematurity <stage 2). Rate of change variables were calculated using the estimates for the random slopes for each subject from mixed models with a random intercept and slope using a first-order autoregressive covariance structure. Log(age) was used because growth rates for fat mass and % body fat are not expected to be linear. Covariates for the body composition analyses included gestational age at birth, birthweight, total kcal/kg days 2–8, and total protein (g)/kg days 2–8, and covariates for the growth factor analyses included sex and gestational age at birth. Analyses were conducted using SAS (Version 9.4, Cary, NC, USA) [18,19].

## 3. Results

### 3.1. Patient Characteristics 

Eighty-three patients had full data available, including at least one body composition measurement while hospitalized, one body composition measurement at term equivalent age, covariate measurements, and retinopathy of prematurity exams. The other 20 patients had incomplete data primarily due to requiring prolonged significant respiratory support or total parenteral nutrition administration, which prevented them from being measured in the air displacement plethysmography pod, or due to death or transfer to another hospital. Table 1 shows a comparison of analyzed and non-analyzed subjects. Non-analyzed patients had more ≥stage 2 retinopathy of prematurity, younger gestational ages at birth, and lower birthweights (*p* < 0.02 for all). Thirty-eight patients had IGF-1, IGFBP3, and adiponectin measured at 7 days and 35 weeks postmenstrual age. One additional patient had retinopathy of prematurity exams and growth factor measurements but was unable to tolerate inpatient body composition measurements due to severity of lung disease; this subject was included in the analysis of growth factors and retinopathy of prematurity severity but not in the analysis of body composition and retinopathy of prematurity severity. There were no differences between the groups of patients with and without growth factor measurements.

Table 2 shows patient characteristics at birth divided by retinopathy of prematurity outcomes, <stage 2 or ≥stage 2. Mean gestational age at birth was 28 0/7 weeks. Fifty-five percent of infants were male and 76% were of non-Hispanic white race. Mean birth weight of all infants was 1092 g (mean birth weight *z*-score −0.08). Infants with ≥stage 2 retinopathy of prematurity had lower median birth weight, length, and occipitofrontal circumference (OFC) (*p* < 0.0001 for all) but not *z*-scores of these measurements (*p* = 0.48−0.98) than infants with <stage 2 retinopathy of prematurity. They also had lower total caloric intake from day of life 2–8 but not lower total protein intake.

### 3.2. Inpatient Growth and Measurements at Term

Median first inpatient body composition measurement occurred at 32 weeks postmenstrual age (range 29–48 weeks), and median last inpatient body composition measurement occurred at 37 weeks postmenstrual age. The term postmenstrual age measurement was defined as each infant’s measurement between 34–42 weeks that was closest to 40 weeks.

Table 3 shows inpatient body composition measurements and rates of change divided by retinopathy of prematurity outcome. At term postmenstrual age, weight for age *z*-score was lower in infants with ≥stage 2 retinopathy of prematurity than <stage 2 retinopathy of prematurity (−0.93 (range −3.94 to −0.30) vs. −0.78 (range −2.71 to −1.58), *p* = 0.026).

### 3.3. Severity of Retinopathy of Prematurity

All infants were evaluated for retinopathy of prematurity. The mean age at most severe retinopathy of prematurity was 35 weeks postmenstrual age. Twenty infants (24%) had ≥stage 2 retinopathy of prematurity, and 63 (76%) had <stage 2 retinopathy of prematurity. Four infants (5%) had retinopathy of prematurity requiring treatment with laser or bevacizumab per recommendations of the Early Treatment of Retinopathy of Prematurity trial [20].

### 3.4. Association of Body Composition and Growth with Retinopathy of Prematurity Severity

Table 4 summarizes the findings of the multivariate logistic regression analysis, which tested for associations between ≥stage 2 retinopathy of prematurity and body composition measurements (fat-free mass, fat mass, % body fat) at term and inpatient rates of change of each of these body composition predictors. Greater gains in all body composition compartments from 32 to 37 weeks postmenstrual age and greater % body fat at term were associated with <stage 2 retinopathy of prematurity (*p* < 0.05 for all) after controlling for gestational age at birth, birthweight, and early total kcal and protein intake. Fat-free mass and fat mass at term were not associated with retinopathy of prematurity severity. At term postmenstrual age, for every additional percentile of % body fat, odds of ≥stage 2 retinopathy of prematurity decreased by 21% (odds ratio, OR, 0.79 (95% confidence interval, CI, 0.64–0.98)). Higher weight-for-age *z*-score at term also decreased the odds of ≥stage 2 retinopathy of prematurity (OR 0.11 (95% CI 0.02–0.57)).

### 3.5. Association of Serum Growth Factors and Retinopathy of Prematurity Severity

In the subset of 39 patients who had full retinopathy of prematurity exams and serum growth factors collected, higher IGF-1 levels at 7 days and 35 weeks postmenstrual age were associated with decreased odds of ≥stage 2 retinopathy of prematurity (OR 0.86 (95% CI 0.75–0.98) and OR 0.9013 (95% CI 0.82–0.99), respectively) when controlling for sex. When gestational age was added to the analysis, the association disappeared (*p* = 0.45 and *p* = 0.58, respectively). Adiponectin and IGFBP3 at both time points were not associated with odds of ≥stage 2 retinopathy of prematurity in any model.

## 4. Discussion

Faster rates of gain of fat mass, fat-free mass, and % body fat from 32 to 37 weeks postmenstrual age and higher absolute term adiposity were associated with decreased odds of retinopathy of prematurity ≥stage 2 among 83 very low birth weight, appropriate for gestational age, preterm infants. These findings imply that targeting improved late somatic growth during the second half of NICU hospitalization may be important for reducing risk for severe retinopathy of prematurity.

Current predictive models identifying infants for retinopathy of prematurity screening typically include postnatal weight gain in the first weeks of life as well as gestational age and birthweight [18,21]. Our findings suggest that growth during the second half of NICU hospitalization should also be considered in these models and that infants with poor late postnatal growth may need closer monitoring than those with appropriate catch-up growth.

Our findings remained significant after adjusting for total caloric and protein intake in the first week of life, factors previously associated with worse ROP outcomes [7,17]. As infants in this cohort with ≥stage 2 retinopathy of prematurity also had lower total caloric intake/kg from days 2–8, this suggests that both early and later periods of nutritional intake and growth during NICU hospitalization are important in ROP development. We have previously shown a link between higher caloric and protein intake in days 2–8 and higher fat-free mass throughout NICU hospitalization, suggesting that early intake impacts later growth, which could impact ROP development [22]. Further study is needed on this topic.

While the relationship between gains in body composition compartments and retinopathy of prematurity severity is novel, gains in these compartments are strongly associated with altered neurodevelopmental and metabolic outcomes. Greater gains in fat-free mass in infancy and early childhood are associated with improved speed of processing and overall cognition, while greater gains in fat mass in childhood increase risk for hypertension and insulin resistance [23,24]. Predictors of adverse body composition status (low lean mass and/or high adiposity) are both nutritional and non-nutritional, with risk factors present both prenatally (such as maternal hypertension) and postnatally (such as nutrient intake, steroid exposure, or degree of illness) [25,26,27,28]. Therefore, monitoring quality of growth, including body composition, is important for multiple outcomes including retinopathy of prematurity.

Our findings also suggest that there may be a differentially protective effect of adiposity as compared to lean mass in the development of more severe retinopathy of prematurity, but further research is needed to determine this relationship. While our adiponectin levels at 35 weeks did not reach statistical significance (*p* = 0.07), it is plausible that adiponectin (positively associated with adiposity and a known anti-inflammatory factor) could mediate such a role. Siahanidou et al. described higher adiponectin levels in infants with greater weight gain in the NICU, and higher adiponectin levels have been associated with less severe retinopathy of prematurity [9,29]. Further research is needed to determine if adiposity is protective against the development of severe retinopathy of prematurity and to determine if elevated adiponectin is a primary mediating factor.

## 5. Conclusions

As this is the first study to examine the relationship between body composition and its changes with retinopathy of prematurity outcomes, these findings require further validation. Ongoing research is needed to further elucidate the differential effects of each of the body composition compartments on retinopathy of prematurity risk. Understanding how growth in these compartments impacts retinopathy of prematurity development may aid clinicians regarding what types of growth to target in infants as they approach NICU discharge and may identify infants in need of closer follow-up for retinopathy of prematurity.

## Figures and Tables

**Table 1:** Characteristics of analyzed and non-analyzed subjects.

In Analysis Group							
	No		Yes			All Subjects	
Characteristic	*N*	Mean ± SD or *n* (%)	*N*	Mean ± SD or *n* (%)	*p*-value	*N*	Mean ± SD or *n* (%)
Gestational Age (Week)	20	26.6 ± 2.9	83	28.1 ± 2.2	0.013	103	27.8 ± 2.4
Birth Weight (g)	20	889 ± 314	83	1092 ± 285	0.006	103	1053 ± 300
≥Stage 2 ROP	15		83		0.005	98	
No		6 (40)		63 (76)			69 (70)
Yes		9 (60)		20 (24)			29 (30)

ROP = retinopathy of prematurity. SD = standard deviation.

**Table 2:** Characteristics of Very Low Birth Weight (VLBW) infants with full data available, divided by ROP outcome.

	ROP <Stage 2			ROP ≥Stage 2			*p*-Value
Characteristic	*N*	Median	Min–Max	*N*	Median	Min–Max	
Sex	63			20			0.97
Female	28 (44%)			9 (45%)			
Male	35 (56%)			11 (55%)			
Gestational Age (Week)	63	29.1	24.6–31.6	20	25.7	22.1–28.9	<0.0001
Birth Weight (g)	63	1190	540–1730	20	705	408–1130	<0.0001
Birth Length (cm)	63	38.0	31.5–44.0	20	33.0	28.5–38.0	<0.0001
Birth OFC (cm)	63	26.5	21.5–29.5	20	22.8	19.0–25.0	<0.0001
Birth Weight Z Score	63	−0.18	−1.22–1.20	20	0.01	−1.63–0.98	0.98
Birth Length Z Score	63	−0.30	−2.00–1.30	20	−0.15	−1.20–1.10	0.48
Birth OFC Z Score	63	−0.40	−1.80–1.50	20	−0.15	−1.40–1.50	0.92
SNAPPE-II Score at Day 7	63	0	0–32	20	5	0–30	<0.0001
Total kcal/kg Days 2–8	63	725.0	539.5–859.2	20	565.9	447.0–807.1	<0.0001
Total Protein (g)/kg Days 2–8	63	25.7	15.3–31.8	20	26.5	18.9–30.0	0.57

ROP = retinopathy of prematurity. OFC = occipitofrontal circumference. SNAPPE-II = Score for Neonatal Acute Physiology with Perinatal Extension-II.

**Table 3:** Anthropometric and body composition measurements and inpatient rates of change, divided by ROP outcome.

	ROP <Stage 2			ROP ≥Stage 2			*p*-Value
	*N*	Median	Min–Max	*N*	Median	Min–Max	
	63			20			
Post-conceptual age at term (week)	60	36.8	34.0–41.9	19	39.4	35.6–41.9	0.0003
Weight at term (g)	59	2490	1816–3980	19	2696	1970–4137	0.19
Weight-for-age *z*-score at term	59	−0.78	−2.71–1.58	19	−0.93	−3.94–0.30	0.026
Length at term (cm)	60	45.0	40.0–51.2	19	45.5	41.0–50.6	0.41
Head circumference at term (cm)	60	33.2	30.5–38.1	19	33.0	30.4–38.3	0.80
Relative weight gain at term (g/kg/day)	59	20.4	9.8–41.2	19	26.8	14.4–63.4	0.0001
Fat-free mass at term (g)	60	2051	1602–2979	19	2194	1647–2994	0.29
Fat mass at term (g)	60	415	172–1076	19	503	215–1143	0.14
% body fat at term	60	17.7	8.4–28.5	19	19.0	8.9–27.6	0.12
Inpatient rate of change: Weight (g/week)	63	172	158–192	20	171	152–180	0.40
Inpatient rate of change: Length (cm/week)	63	1.00	0.91–1.11	20	0.96	0.86–1.06	0.0007
Inpatient rate of change: OFC (cm/week)	63	0.89	0.85–0.96	20	0.85	0.80–0.91	<0.0001
Inpatient rate of change: FFM (g/week)	63	151	138–167	20	145	128–159	0.0007
Inpatient rate of change: FM (g/log(week))	63	2369	2297–2509	20	2358	2245–2486	0.55
Inpatient rate of change: %BF (%/log(week))	63	61.0	59.6–63.0	20	61.1	59.4–62.4	0.63

FFM = fat free mass. FM = fat mass. BF = body fat. ROP = retinopathy of prematurity.

**Table 4:** Association between ≥stage 2 ROP and body composition variables’ measurements at term postmenstrual age or inpatient rates of change, unadjusted and adjusted for gestational age at birth, birthweight, total kcal/kg days 2–8, and total protein (g)/kg days 2–8.

	Unadjusted		Adjusted	
Variable	OR (95% CI)	*p*-Value	OR (95% CI)	*p*-Value
Fat-free mass (FFM) at term (g)	1.0008 (0.9993, 1.0022)	0.29	0.9984 (0.9958, 1.0010)	0.24
Fat mass (FM) at term (g)	1.0016 (0.9995, 1.0038)	0.14	0.9961 (0.9919, 1.0003)	0.07
% body fat at term	1.0908 (0.9782, 1.2163)	0.12	0.7889 (0.6367, 0.9774)	0.03
Inpatient rate of change: FFM (g/week)	0.8691 (0.7928, 0.9527)	0.003	0.6928 (0.6367, 0.9774)	0.009
Inpatient rate of change: FM (g/log(week))	0.9964 (0.9851, 1.0079)	0.54	0.9622 (0.9365, 0.9886)	0.005
Inpatient rate of change: %BF (%/log(week))	0.8371 (0.4119, 1.7012)	0.62	0.0721 (0.0101, 0.5162)	0.009
Weight-for-age *z*-score at term	0.5060 (0.2696, 0.9498)	0.034	0.0450 (0.0042, 0.4877)	0.011

BF = body fat. OR = odds ratio. CI = confidence interval.
